# Study on exercise muscle fatigue based on sEMG and ECG data fusion and temporal convolutional network

**DOI:** 10.1371/journal.pone.0276921

**Published:** 2022-12-01

**Authors:** Dinghong Mu, Fenglei Li, Linxinying Yu, Chunlin Du, Linhua Ge, Tao Sun

**Affiliations:** East China University of Technology, Nanchang, Jiangxi, China; Newcastle University, UNITED KINGDOM

## Abstract

**Background:**

Muscle fatigue is a crucial indicator to determine whether training is in place and to protect trainers.

**Purpose:**

To make full use of morphological information of surface EMG and ECG signals in the time domain, a new idea and method for the fatigue assessment of exercise muscles based on data fusion is proposed in this paper.

**Methods:**

sEMG and ECG time series with the same length were obtained by signal preprocessing and sequence normalization, feature extraction of sequence tenses was realized by a deep learning network based on sequential convolution and signal fusion model of muscle fatigue evaluation was established by D-S evidence theory.

**Experiment:**

Thirty volunteers were recruited and divided into three groups. ECG signals and sEMG signals at the biceps brachii of the right upper limb were monitored in a 20-minute exercise cycle.

**Results:**

The prediction result of TCN based on time domain signal is better than the commonly used KNN and SVM recognition algorithm, and the recognition accuracy of relaxed, excessive and fatigue by D-S fusion was 89%, 86%, 88.5%. The accuracy was 0.9055, 0.9494 and 0.9269, respectively. The recall rates of the three conditions were 0.9303, 0.9570 and 0.9435. The F-score of the three conditions was 0.8911, 0.8764 and 0.8837, respectively.

**Conclusion:**

Based on time series and time series convolutional network, sEMG and ECG fusion of motor muscle recognition method can better distinguish different state information and has certain practical value in the fields of muscle evaluation, clinical diagnosis, wearable devices and so on.

## 1. Introduction

In recent years, the detection of surface electromyography (sEMG) and electrocardiogram (ECG) has been developed rapidly. These two techniques have the advantage of directly reflecting neuromuscular activity [1], and have been widely used in medicine, clinical diagnosis and sports science. sEMG is a kind of bioelectrical signal generated by the neuromuscular system during the contraction of skeletal muscle. It is formed by the comprehensive superposition of muscle motor units on the skin surface. The signal changes are related to muscle activity level and functional status. It is often used as a physiological signal to assess muscle fatigue. ECG contains a large amount of functional state information of human motor nerves [2]. It is a comprehensive reflection of heart activity on the body surface [3]. It is widely used in muscle state assessment, emotion estimation, clinical diagnosis, body state assessment and other fields.

sEMG and ECG are nondestructive, rapid and convenient monitoring methods, which have been reported in the field of body condition monitoring in sports science. Chang et al. [1] developed a wireless sEMG muscle monitoring system. Wei et al. [4] established the correlation between muscles and gestures by arranging sEMG sensors in the upper limbs, and achieved good recognition results. Costa et al. [5] proposed a standard scheme for detecting muscle sEMG signal Moniri et al. [6] proposed an adaptive algorithm to monitor the sEMG signal of the body, which provides technical support for the development of wearable devices. At the same time, ECG has also been reported in the field of muscle state and motion analysis. Thompson et al. [7] in order to eliminate the problem of motion artifacts in ECG, a new feature extraction algorithm is proposed to improve the stability of ECG model. Parr et al. [8] through the retrieval of electronic database, determined the introduction standard of 38 indexes of ECG, and established the mapping relationship between ECG and body learning and movement. These studies show that both sEMG and ECG can reflect many indicators of the human body. In recent years, there have been many research reports on the fusion of the two monitoring technologies. Li et al. [9] used the signal fusion of sEMG and ECG to establish a prediction model for the motion intention of upper limb amputation, so as to provide technical support for the auxiliary development of intelligent wearable devices. Wei et al. [10] used sEMG and ECG features to recognize the gait of 7 healthy volunteers. Based on the mean power frequency (MPF) and integrated electromyography (iEMG) of sEMG and the slope change of ECG, a support vector machine library was established for classification. Finally, the accuracy of gait phase recognition remained above 96%. These research results show that the signal fusion technology of sEMG and ECG has great application prospects in motion state recognition and fatigue evaluation.

The traditional analysis method is based on sEMG or ECG signal preprocessing to obtain the characteristic values of the signal, such as integrated electromyography (iEMG), root mean square (RMS), median frequency (MF) and MPF. These characteristic values are used to establish the prediction model [10]. However, these characteristic parameters have great correlation and redundancy [6]. The models based on these eigenvalues often have low accuracy and poor robustness. So we need to find another way to study sEMG [11] and ECG signals. In recent years, with the rapid development of deep learning algorithm, feature self-learning becomes possible, which is often used in image recognition, speech recognition and other fields. Several common sequential data processing algorithms such as convolutional neural networks (CNN) [12], recurrent neural network (RNN) [13], long short term memory (LSTM) [14] have been widely used. After the preliminary research, sEMG and ECG signals are a set of one-dimensional unstructured data, from which time series spectrum can be proposed and input into deep learning network to establish sEMG and ECG fusion analysis model [15]. sEMG and ECG signals are rich in a variety of information of the body, and their changes are diverse and complex. A learning network that can comprehensively learn the morphological changes in the time series is needed. The temporal convolutional network (TCN) can flexibly expand the range of receptive field through the expansion spinner, so as to obtain longer time series information. Compared with the way of using pool layer and deepening the network layer to increase the receptive field in the general convolution network, it has less risk of losing characteristic information and less computation. At the same time, compared with LSTM and RNN, it has faster training speed and stronger ability to capture temporal dependencies.

Therefore, aiming at the problem of poor robustness of the muscle fatigue evaluation model based on signal features, this paper proposed a sEMG and ECG muscle fatigue identification method based on time series and TCN. This method directly established a single cycle time series of sEMG and ECG signals and input them into the TCN model. Using the feature grasping ability of TCN to the time series, it completed the extraction of signal morphological features, and established the muscle fatigue prediction models of sEMG and ECG respectively. The classical D-S evidence theory method was used to fuse the models, and finally the overall prediction model was obtained. The statistical parameters of each prediction model were compared. This research will provide technical support for the fusion application of sEMG and ECG.

## 2. Materials and methods

### 2.1. Data collection

**Fig 1:**
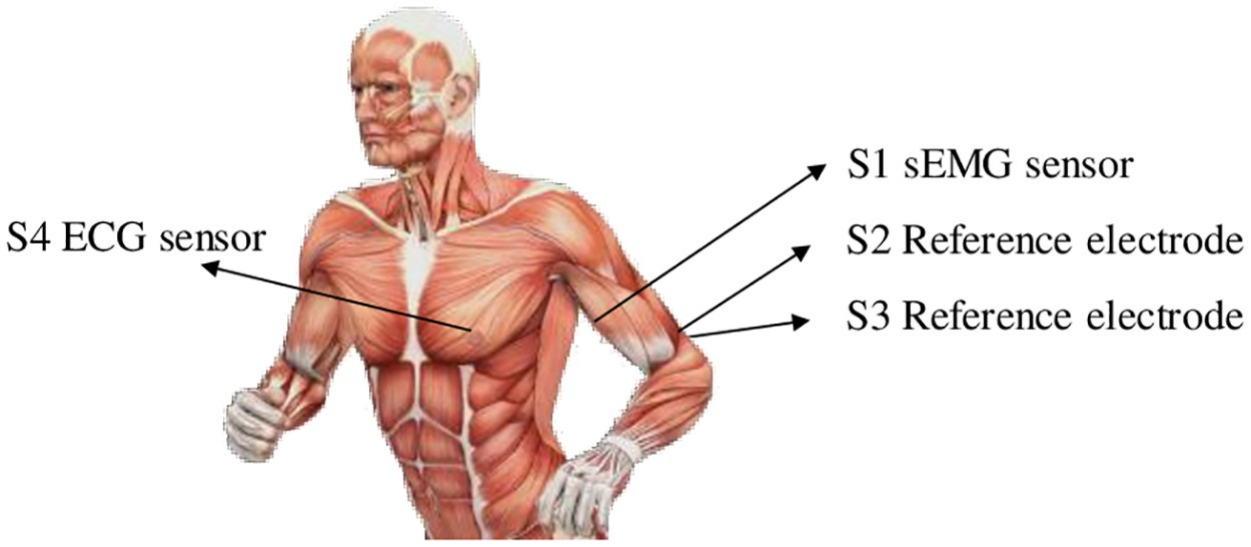
Upper limb test process and sensor position.

This section describes the data collection and interpretation analysis methods. In order to verify the improved fusion algorithm proposed in this paper, 30 healthy volunteers (15 male and 15 female) were recruited for the study, aged (22.4±1.5) years old, height (173.2±5.8) cm, weight (65.3±7.2) kg, body mass index was 22.7±1.8. All subjects were healthy without any diseases. The data were collected in the gymnasium of East China University of Technology. Before the experiment, all subjects were familiar with the experiment plan and procedure and signed the volunteer informed consent. The whole experiment had been reviewed by the Ethics Sub-Committee of Human Medical Experiment of East China University of Technology (No. 2021EC(R)017). Subjects did not exercise more than twice a week in the 3 months prior to the trial and did not exercise vigorously in the 48 hours prior to the trial. SEMG signal acquisition equipment adopted sEMG signal acquisition module (EMGduino 8-channel EMG acquisition module, Hangyi Biology, China). The ECG was collected by KF12 multi-parameter monitor (Hunan Kefu Medical Technology Development Co., China), which has its own rechargeable lithium battery and can print data results automatically. According to the physiological structure of human body, ECG signal synchronously collected the sEMG signal of right upper limb biceps brachii and motion capture data of right upper limb of each subject. The sampling frequency of sEMG signal was 1 kHz. The positions of sEMG and ECG sensor are shown in Fig 1. S1 was the sEMG signal sensor at the biceps brachii of the right upper limb, S2 and S3 were the signal reference electrodes of sEMG. S4 was the ECG sensor.

According to the physiological structure of the human body, ECG signal and sEMG signal of right upper limb biceps brachii were collected synchronously. The laboratory temperature was 20°C±3°C. Clean the skin surface of the subject with scrub cream and 75% alcohol cotton, remove the oil on the skin surface, etc., and reduce the interference of skin impedance. According to the principle of anatomy, the sEMG sensor was attached to the humps of the three muscles. To stick more closely with the muscles and obtain more accurate signals, a small amount of water can be attached to the one-time sEMG. After the sEMG sensor was fitted, wrapped the sensor around the test site with an adhesive cloth to prevent the electrode adhesive from falling off during the experiment.

The subjects were divided into three groups of 10 people in each group. According to the plan, the prescribed training was carried out. Set a 20-minute training cycle. Ten subjects completed one training cycle as a group. Each experiment lasted 60 minutes, and a total of 10 experiments were conducted. The first group completed the test, the second group continued the test, and the first group rested. During each training session, subjects sat in front of the lab table with their back straight at 90° and their left arm naturally lowered. During the experiment, sEMG signal was collected for 5s without weight, and then the experimenters placed the dumbbell in the hands of the subjects. The subjects slowly lifted the dumbbell, keeping the stability of the upper arm of the right hand. Subjects reported how they felt about their fatigue state by lowering dumbbells every 120 seconds according to the RPE scale [16] and marked their fatigue state value at this time (relaxed: -1, transition: 0, fatigue: 1).

The sEMG and ECG signals of the three states were marked and saved, and the corresponding training time was recorded. 600 groups of sEMG and ECG signals were obtained in each group. According to the corresponding fatigue value, each data was divided into three states, a total of 300 sEMG and 300 ECG data. After the experiment, 6000 sEMG and ECG data were collected respectively. All subjects underwent the same signal acquisition and analysis process.

CV is a method that divides the data set into two groups as ‘training’ and ‘sample’ for supervised learning. 10-fold CV firstly obtains 10 subsets having equal number of members from the data set and members are randomly shared by the subsets. For one classification process, each of the subsets is used as a training set and the other subsets as a sample set. So, the resultant prediction accuracy rate is calculated by the average of 10 prediction accuracy rates.

### 2.2. Signal preprocessing

ECG and sEMG signals are highly contaminated by environmental interference, such as 50 Hz powerline electromagnetic interference. The ECG signal was filtered by a third-order Butterworth low-pass filter. The EMG signals were pre-amplified using an instrument amplifier with gain of 5 and then filtered using second-order stopband analog filters with cutoff frequencies of 60 Hz and 160 Hz to minimize the effects of electromagnetic interference and artifacts. Finally, the signal was amplified 318 times to make it usable. So, the total amplification gain is 1590.

sEMG signals are non-stationary in nature. The most appropriate way to study these signals is to use time-frequency methods such as wavelet decomposition. This method was developed by Mallat in 1980 for data compression in image coding. Wavelet decomposition represents information extracted from the time domain and frequency domain of a signal based on the application of a unique parent wavelet transform. Finally, sEMG and ECG single—period signals are obtained by periodic segmentation and periodic averaging.

**Fig 2:**
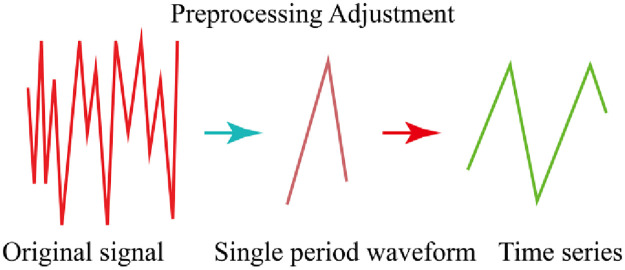
The extraction process of time series.

Signals of different individuals are not consistent, so the single-period waveform obtained is also different in data length. However, deep learning networks generally require features of consistent input length, so it is necessary to normalize the time series length of single-period signals. There are two commonly used signal normalization processing methods: tail zeroing and multi-sampling rate resampling, but the former will lead to a large amount of redundant information in short period time series. The latter may lose some important information. Therefore, in order to maintain the original form and information integrity of time series, the normalization of single-period waveform in this paper is as follows: Firstly, a uniform length of time series is set so that it can accommodate at least one single-period signal; Secondly, the single-period signal segmented by the same sample is supplemented at the end of the sequence until the set sequence length is reached. The extraction process of time series is shown in Fig 2.

### 2.3. Morphological feature self-learning based on TCN

TCN is a network that can be used to deal with timing problems. For a long time series, traditional CNN cannot capture the dependent information in the series well in a limited level due to the limitation of the convolution kernel size. TCN uses a new network structure to solve the defect of CNN in dealing with timing issues. TCN is composed of multiple residual blocks in series.. In the residual block, there are two levels of dilatational causal convolution with the same parameters, and the linear rectifier function is used as the activation function after the weight normalization. In addition, regularization is performed after each convolution layer to prevent overfitting. 1×1 convolution is used to ensure that the dimensions of the input and output tensors are consistent in the process of the identity mapping with residual connection.

#### 2.3.1. Causal convolution

Causal Convolutions with one-dimensional full convolutional network structure are used in TCN to transform the conventional two-dimensional convolutional neural network into the application of one-dimensional time series data, Therefore, sEMG and ECG time series which are both one-dimensional time series can be processed. Meanwhile, in the causal convolution, the output of the current node is only related to the input of the current node in the previous layer and the input before it, so the dependence between the current characteristic point in the time series and the morphological features in the past time can be reflected. In other words, assume that the convolution kernel size of the one-dimensional time series input (*x*_0_, *x*_1_, *x*_2_, ⋯, *x*_*t*_, ⋯, *x*_*n*_) from the upper layer is *k*×1, then the causal convolution of the current layer on the time node *t* is:

$$x_{j,t}^{\left(l\right)}=\sigma \left[\left(x_{i,t-k+1}^{\left(l-1\right)}\cdots ,x_{i,t}^{\left(l-1\right)}\right)*W_{ij}^{\left(l\right)}+b_{j}^{\left(l\right)}\right]$$ (1)

Where * is the convolution operation, $x_{j,t}^{\left(l\right)}$ is the value of node *t* after the *j*-th convolution kernel operation on *l* layer, $W_{ij}^{\left(l\right)}$ is the *j*-th convolution kernel on *l* layer, $b_{j}^{\left(l\right)}$ is the bias value, and σ is the activation function. It can be seen from the formula (1) that causal convolution will not add the future value of the sequence into the operation.

#### 2.3.2. Dilatative convolution

**Fig 3:**
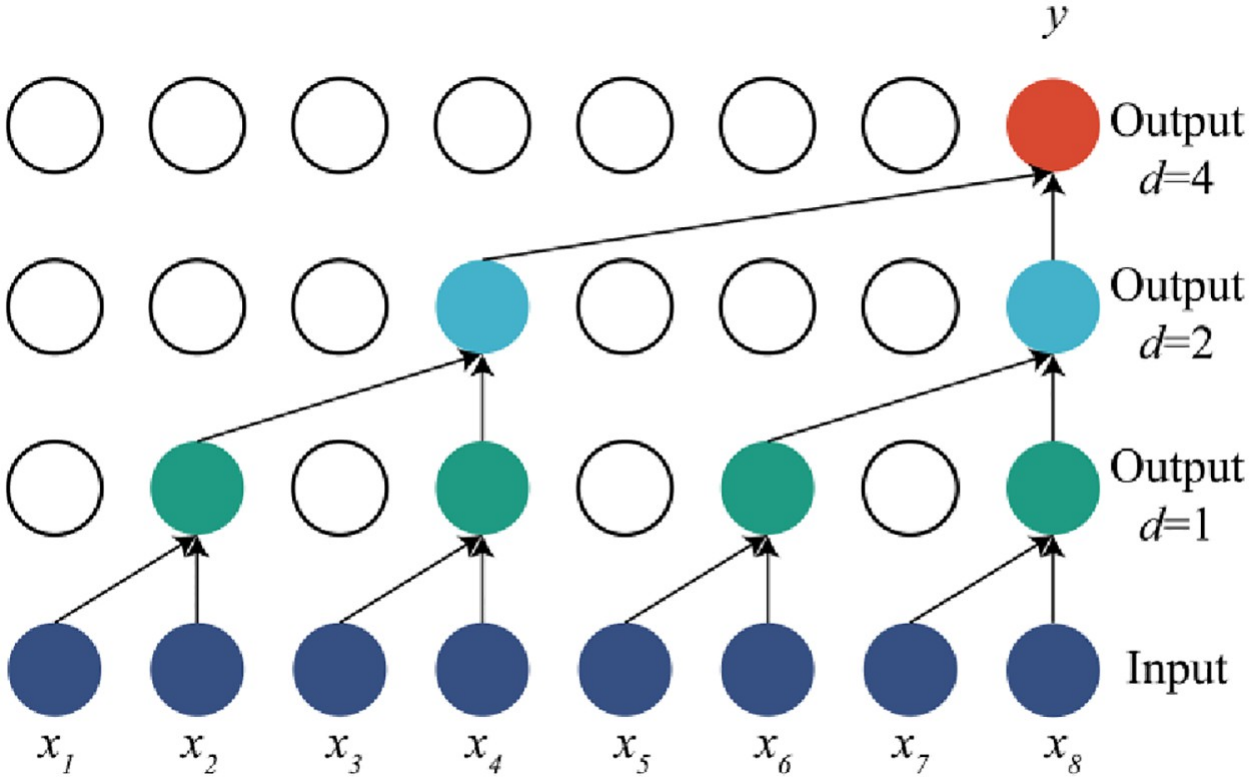
Dilation causal convolution, *k* = 2, *d* = [1,2,4], step size is 1.

In order to carry out convolution processing on time series and capture more morphological changes on time series, the information needs to expand the receptive field of neural nodes. To expand the receptive field in standard convolutional networks, there are generally ways such as increasing the depth of network layer, expanding the size of convolution kernel, pooling and increasing step size. However, this may lead to a large increase in computation and the loss of important feature information in time series. In the process of convolution operation, dilatative convolution can achieve exponential growth of the receptive field while retaining the characteristics of time series by injecting cavities into the standard convolution kernel. As shown in Fig 3. On the basis of causal convolution, holes are added to the convolution kernel according to the expansion rate to realize the dilated convolution kernel. When *d* = 1, the size of the convolution kernel is consistent with the standard convolution kernel of 2×1. In one-dimensional convolution, the receptive field expands to 4. When *d* = 4, the receptive field of the node pair sequence expands twice again.

If the input time series is defined as layer 0, *RF*_(*m*,*n*)_ is the size of the receptive field of nodes in layer *m* at layer *n*. Then, on the premise that the step size is 1, the size of the receptive field of each layer in the expansion convolution is as follows:

$$RF_{\left(m,n\right)}=1+\sum_{i=n+1}^{m}d_{i}\left(k_{i}-1\right)(m,n\in N,m>n;k,d\in Z+)$$ (2)

Where *k*_*i*_ and *d*_*i*_ are respectively the size and expansion rate of the convolution kernel during the expansion convolution of layer *i*-1. If the size *k* of the convolution kernel of each layer remains unchanged, the size of the receptive field of nodes in each convolution layer at layer 0 after simplification is:

$$RF_{\left(m,0\right)}=1+(k-1)\sum_{i=1}^{m}d_{i}\;(m,k,d\in Z+)$$ (3)

**Fig 4:**
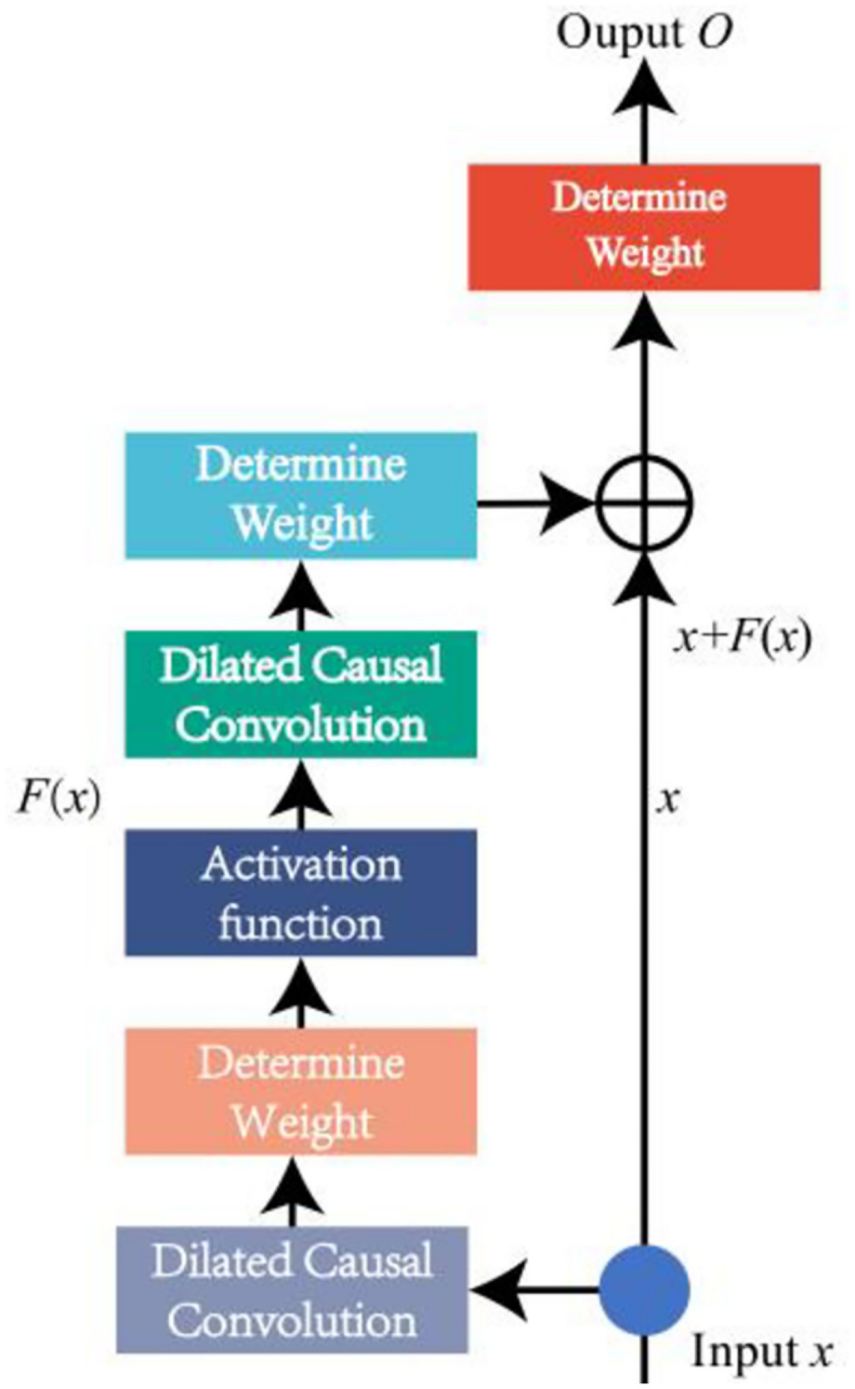
General structure of residual block.

Residual connection is widely used in deep neural networks, which can solve the problems of gradient disappearance and gradient explosion caused by too many layers. TCN generally uses progressive increasing *d* value to expand the receptive field of neural nodes, and the network depth will increase accordingly. Therefore, the introduction of residual connection can make the network more stable. The general structure of residual block is shown in Fig 4, whose output O contains the output of *F* and input *x* added through identity mapping:

o=σ(F(x)+x) (4)

**Fig 5:**
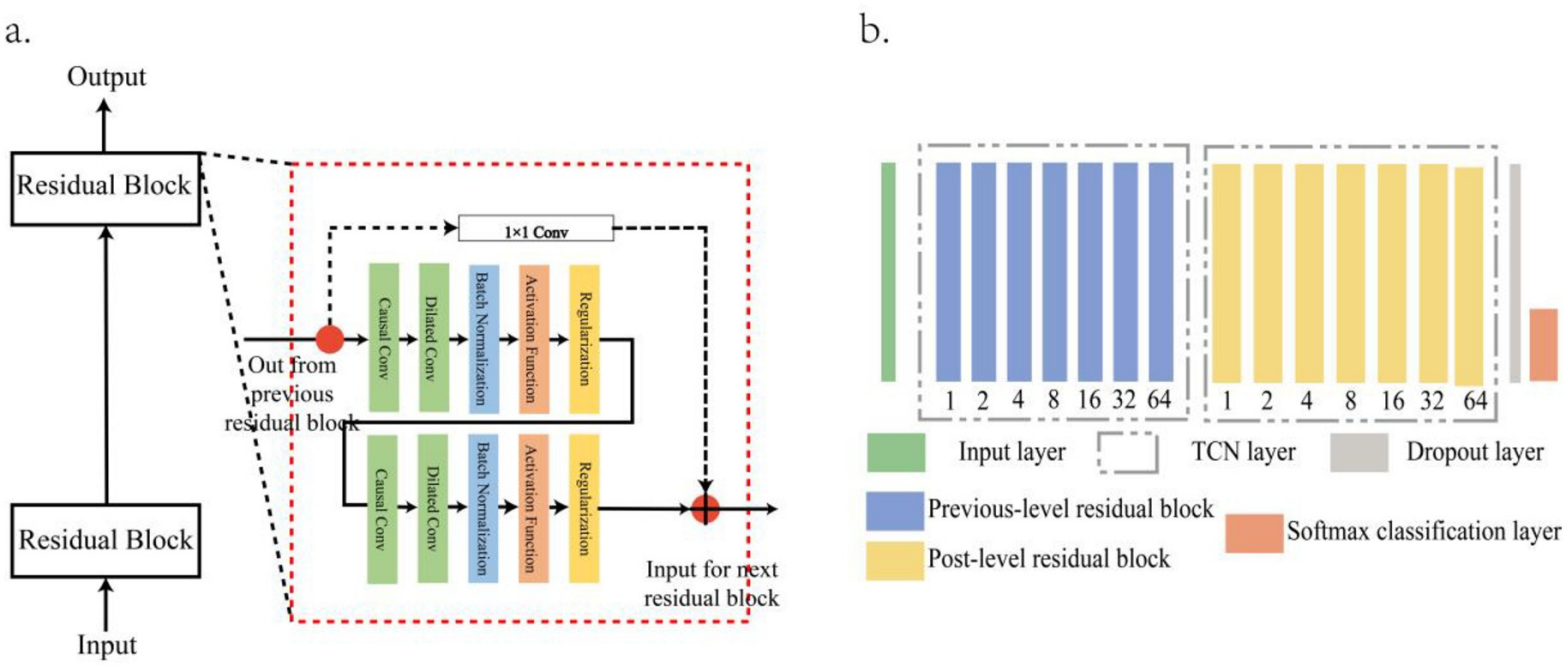
TCN structure, a. The overall structure, b. The network structure.

The overall structure and network structure of TCN are shown in Fig 5, The structure consists of several residual blocks connected in series, each of which includes causal convolution and dilated convolution.

### 2.4. TCN time series signal prediction model

**Fig 6:**
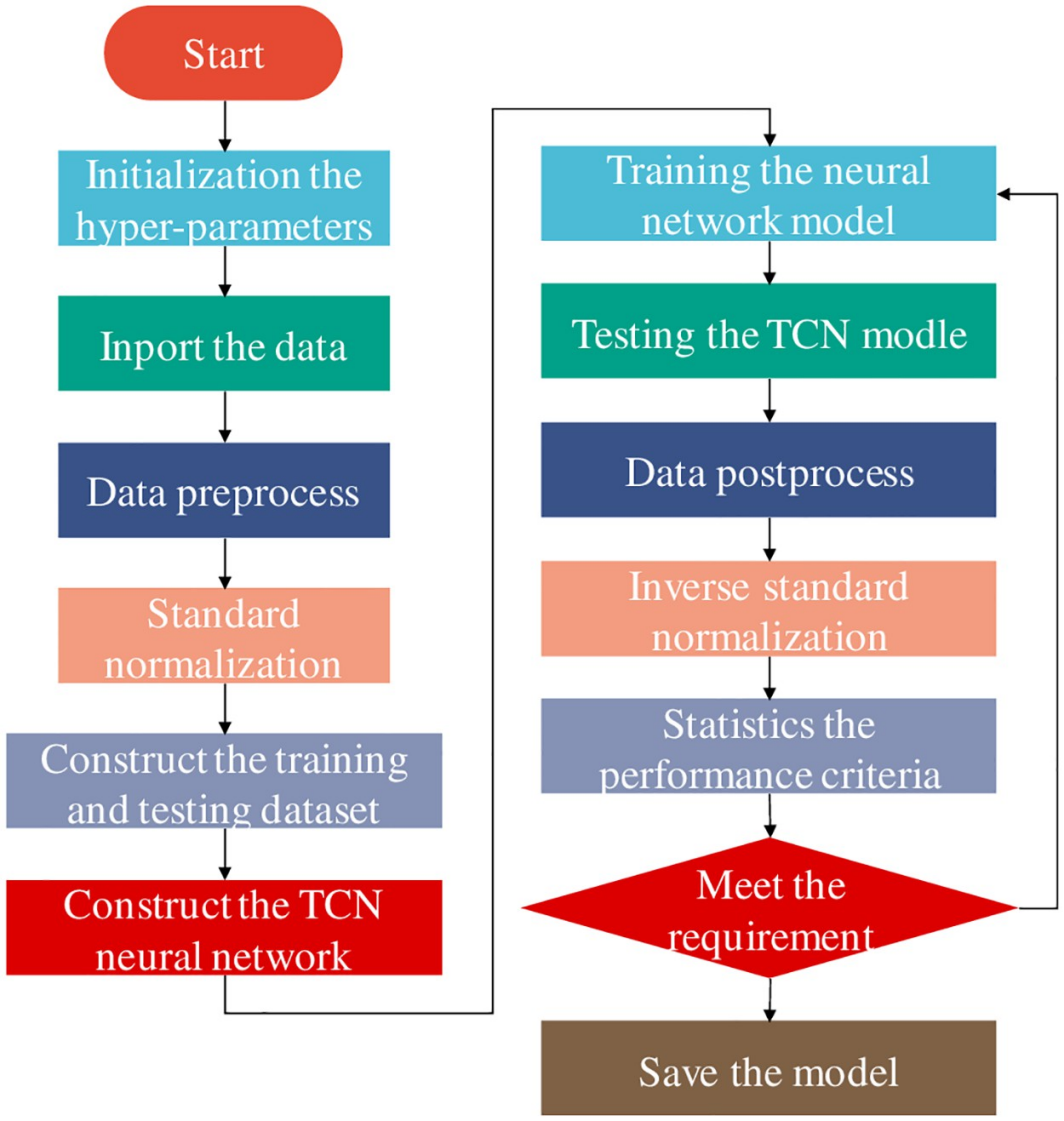
Network structure of TCN time series prediction model.

In this paper, a timing signal prediction model was established based on TCN, as shown in Fig 6. In the network structure of this model, two levels of TCN were stacked to make the receptive field larger and the network more stable. The dimension of the input time series was 800×1. After the test, the convolution layer used a 5×1 filter, the sliding step was 1, and the number of filters was set to 32 and 64 in the front and back stages, respectively. The expansion rate (the *d* in Formula 2 and 3) in the TCN residual block is set as [1,2,4,8,16,32,64], and the zigzag structure is repeated in the two-stage TCN. The receptive field of the front stage for time series was 509, while the receptive field of the back stage could be increased to 1017 by using two-stage TCN. Stacked TCN could achieve a large receptive field through a few layers of networks, greatly reducing the computational cost compared with traditional networks such as CNN, and also had good feature information capture capability for a long time series.

After the TCN time series evaluation of the two monitoring techniques, the two prediction results were fused at the decision level. D-S evidence theory method is suitable for multi-source heterogeneous information fusion and can realize the fusion and complementarity of multi-source heterogeneous data to form consistent and comprehensive maintainable data. D-S evidence theory was used with reference to [17]. According to this theory, there are three states as data fusion propositions to judge muscle states. The muscle status after TCN was detected by sEMG and ECG. D-S theory constructs the corresponding basic probability distribution function for these evidences with a credibility. A basic probability distribution and the corresponding classification framework are synthesized into an evidence body. Use Dempster’s merger principle [18] to merge each evidence body into a new evidence body. After 10-fold cross-validation, the reliability of sEMG and ECG assignments is sEMG: 0.591, ECG: 0.409.

**Fig 7:**
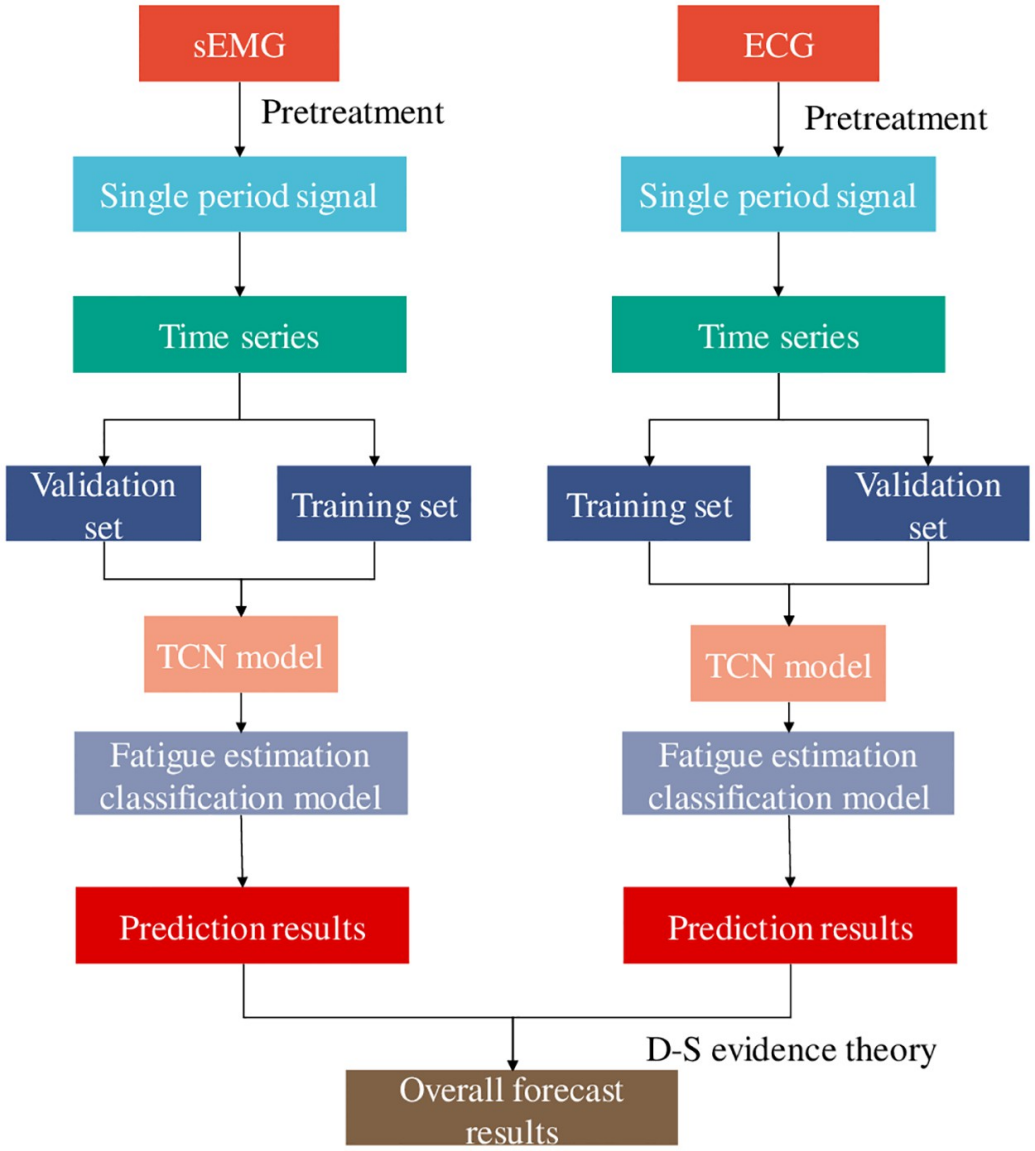
Fatigue evaluation model flow based on TCN and data fusion.

The model established by TCN combined with D-S evidence theory is referred to as sEMG-ECG-TCN, Fatigue evaluation model flow based on TCN and data fusion is shown in Fig 7. In order to compare the prediction results of fused data, sEMG prediction models were also established, namely sEMG-TCN, ECG-TCN, sEMG-KNN and sEMG-SVM. In order to compare the performance of ECG-KNN and ECG-SVM, common method models, sEMG-ECG-KNN and sEMG-ECG-SVM, were established. The establishment method was referenced in [19]. In order to evaluate the advantages and disadvantages of the fusion model, Accuracy, Recall and F-Score were used to evaluate the overall performance of the model. The detailed calculation method is as follows:

$$Accuracy=\frac{TP+TN}{TP+TN+FP+FN}$$ (5)

Where *TP* is the number of True positives Negatives to identify, *TN* is the True positives Negatives to identify, and *FP* is the number of False positives Negatives to identify, *FN* is the sample number of Flase Negative recognition.


$$Precision=\frac{TP}{TP+TN}$$ (6)

$$Recall=\frac{TP}{TP+TF}$$ (7)

$$F\text{-}score=2\times \frac{precision\times recall}{precision+recall}=\frac{2TP}{2TP+FP+FN}$$ (8)

The whole project was written in Python with Tensorflow 2.3.1 and Sklearn 0.22.2 packages as the core, supplemented by Numpy and Pandas. Drawing using Origin 2018.

## 3. Result

### 3.1. sEMG and ECG signal analysis

Python 3.0 was used to preprocess the collected sEMG signals. The time window was set as 1000 points, and the distance of each window movement was set as 200 points. The eigenvalues of the subjects in three states were calculated respectively. Each parameter index was calculated by the following formula: ECG_mean_ represents the average ECG power, sEMG_RMS_ represents the root mean square (RMS) of sEMG, sEMG_iEMG_ represents the integrated EMG value of sEMG time domain index, sEMG_*ZC*_ represents the zero crossing rate (ZC) of sEMG signal. sEMG_ARV_ represents the average rectified value(ARV) of sEMG. ECG_LF_ represents the power in the low frequency segment (0.04–0.15 Hz) of the ECG sequence, and ECG_LF/HF_ represents the power ratio in the low and high frequency bands of the ECG sequence. Other parameters are calculated as follows:

$${\text{ECG}}_{\text{mean}}=\frac{1}{M}\sum_{i=1}^{M}\left(RR_{i}\right)$$ (9)

$${\text{sEMG}}_{\text{RMS}}=\sqrt{\frac{1}{N}\sum_{n=1}^{N}y_{n}^{2}}$$ (10)

$${\text{sEMG}}_{\text{iEMG}}=\sum_{n=1}^{N}\left|y_{n}\right|$$ (11)

$${\text{sEMG}}_{ZC}=\sum_{n=1}^{N}\left|\text{sign}\left(y_{n}\right)-\text{sign}\left(y_{n-1}\right)\right|$$ (12)

$${\text{sEMG}}_{\text{ARV}}=1/T\int_{t}^{t+T}|\text{EMG}(t)|\text{d}t$$ (13)

**Fig 8:**
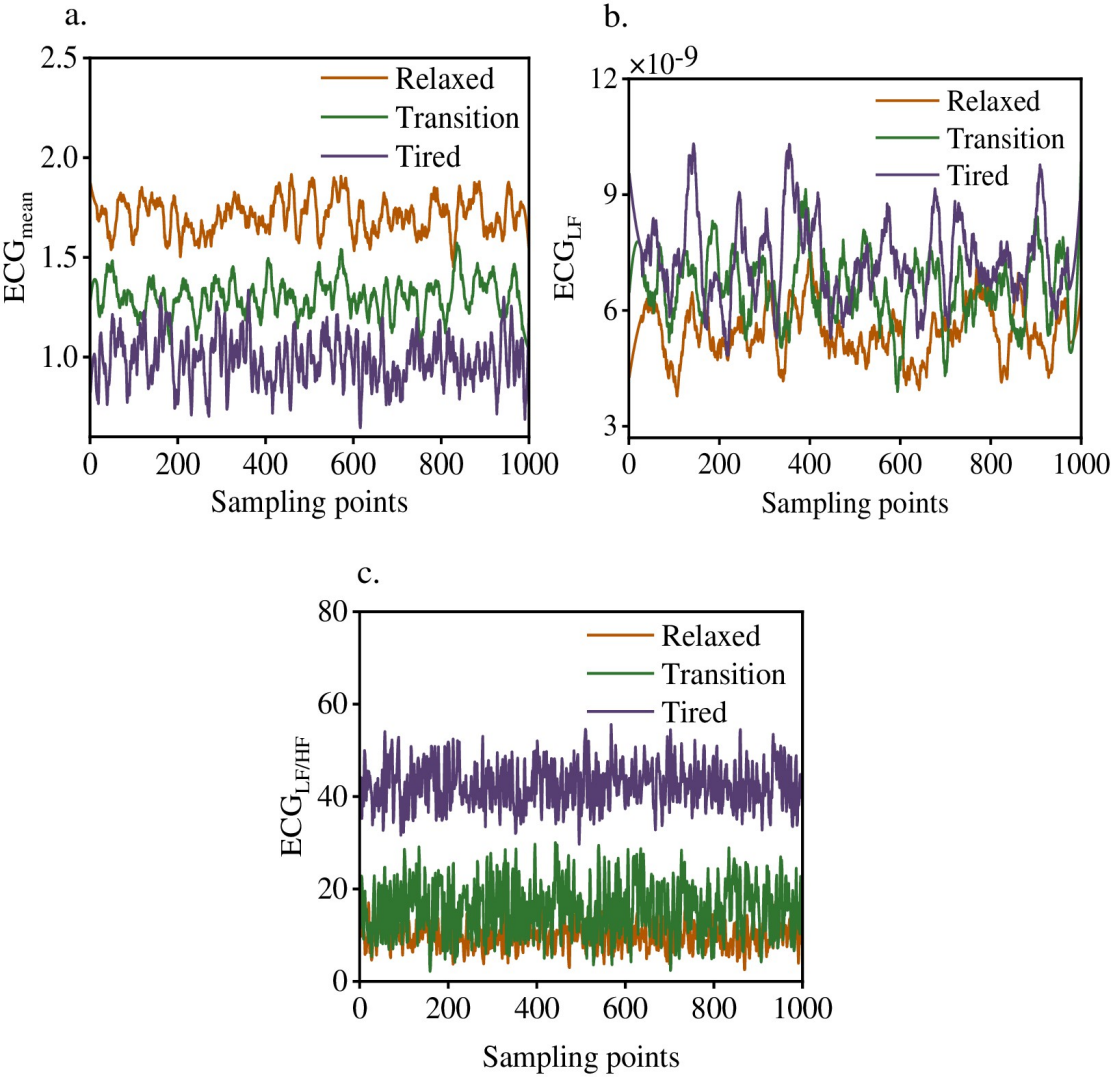
ECG signal characteristic value.

**Fig 9:**
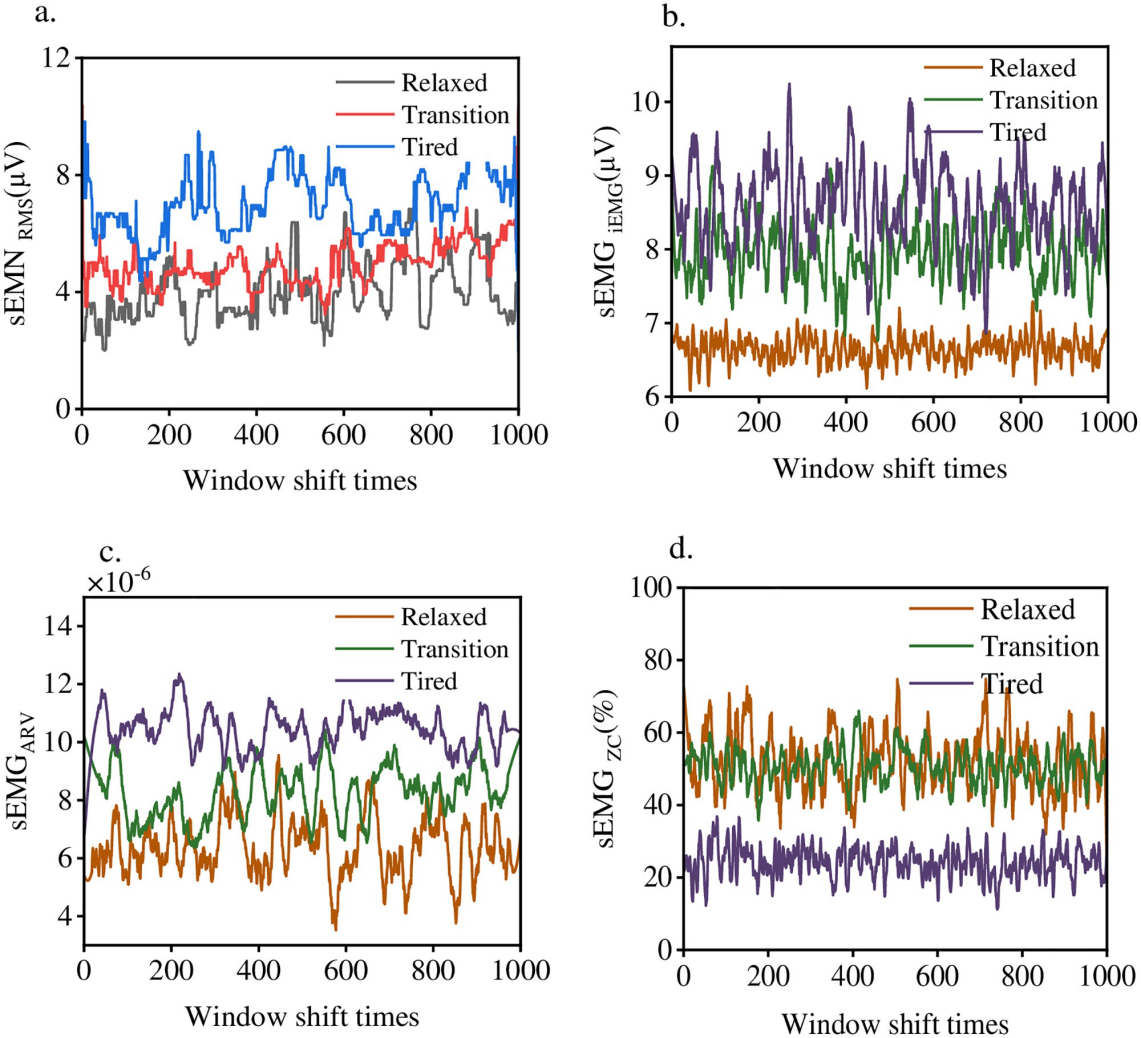
Time domain eigenvalue variation.

At present, a large number of studies have found that with the deepening of fatigue degree, the characteristics of sEMG signal in time domain, frequency domain and eigenvalue will change to varying degrees [20]. Since this paper was based on the model of time sequence signals, only the changes of time domain features were discussed. ECG signal characteristic values are shown in Fig 8. ECG_mean_ decreased gradually with the deepening of movement. ECG_LF_ and ECG_LF/HF_ did not differentiate the three states well. The classic RMS, iEMG, ARV and ZC characteristic values of sEMG with muscle state is shown in Fig 9. RMS, iEMG and ARV increased while ZC decreased when the muscle entered the transition state from relaxation. However, RMS and iEMG signals overlapped in the process of change and could not distinguish the relaxed state from the transition state well. The sensitivity of ZC was not strong, and the ZC features of easy and transitional states cannot be intuitively distinguished. When the transition state enters the fatigue state, RMS is improved more, and fatigue and transition state can be intuitively distinguished. The sensitivity of ZC was strong. When entering the fatigue state, ZC decreased significantly and did not overlap with the relaxed and transitional state. The statistical results were similar to those reported in [21]. The reason for this is that, in weight-bearing condition, the muscle recruits more fibrous motor units, thus generating more action potentials. Influence the change of each index. However, it is difficult to establish evaluation indexes through simple classification model because of the different degree of influence on individuals.

In conclusion, sEMG has many time-domain features, some of which show good sensitivity to transition and fatigue states, while some indicators may overlap in the process of change. Most of the characteristics will increase with the degree of fatigue. This is very unfriendly to the establishment of quantitative models, because there are a large number of collinearity factors in the input, and the established model has poor robustness. Therefore, it is not accurate to build a prediction model based on a single or a small number of time domain features. Current research is focused on building more features into prediction models, which does improve accuracy, but models are not universal. Different movements or monitoring positions require changes in different types of features. This is consistent with what was reported in literature [22].

**Fig 10:**
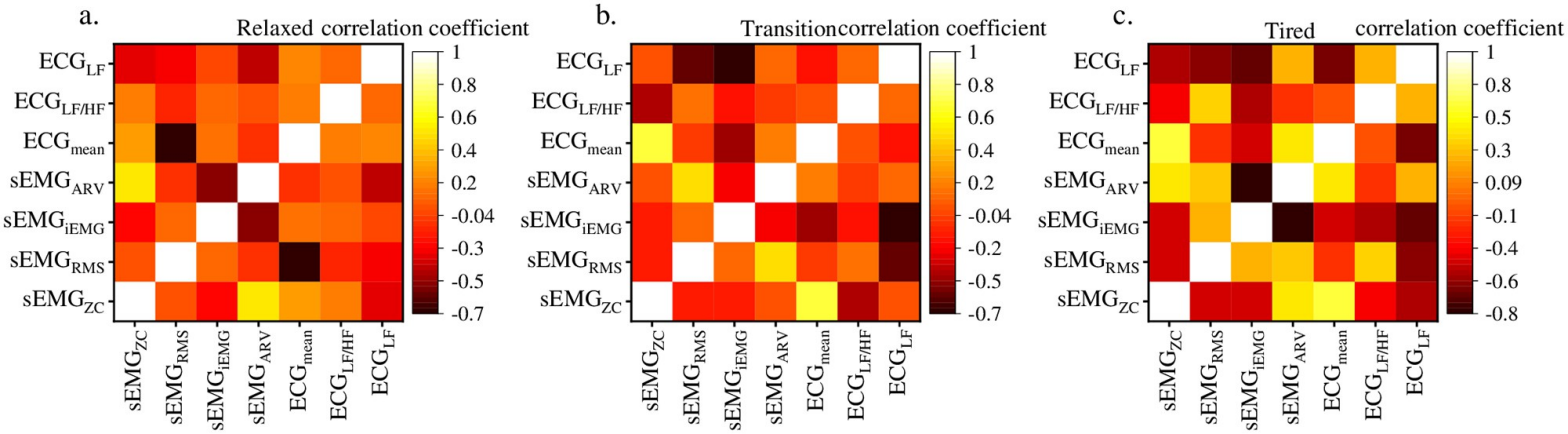
Correlation coefficients of sEMG and ECG time domain eigenvalues, a. Relaxed, b. Transition, c. Tired.

The correlation between sEMG and ECG signals is always used to quantify the effect of artifact removal in ECG signals. Numerous studies have shown that there are artifacts in sEMG and ECG signals. In order to obtain accurate ECG signals, sEMG signals should be used to remove artifacts in ECG signals. If the correlation between the two is reduced, the ECG signal independence is better. In data fusion analysis, the less correlation between the two inputs, the better. Therefore, the correlation between the time-domain eigenvalues of sEMG and ECG signals was analyzed here, in an attempt to analyze the factors affecting muscle fatigue classification from the signal level. The correlation between sEMG and ECG time domain eigenvalues is analyzed, and the correlation coefficient thermal diagram between each eigenvalue is listed to show the relationship between the time domain eigenvalues more intuitively (Fig 10). The lighter the color, the stronger the positive correlation, and the darker the negative correlation. In the relaxation stage, the negative correlation between ECG_mean_ and sEMG_RMS_ was strong, reaching -0.67, while the positive correlation between sEMG_ARV_ and sEMG_ZC_ was strong, reaching 0.5. In the transitional stage, the negative correlation between ECG_LF_ and sEMG_iEMG_ was strong, reaching -0.65, while the positive correlation between ECG_mean_ and sEMG_zc_ was strong, reaching 0.84. In the fatigue stage, the positive correlation between ECG_mean_ and sEMG_zc_ was strong, reaching 0.66. Meanwhile, it should be noted that the negative correlation between sEMG_ARV_ and sEMG_iEMG_ was particularly strong, reaching -0.81. From these analyses, both sEMG and ECG time domain indexes were correlated to a certain extent. The model based on a single index such as sEMG or ECG had a high data redundancy due to the correlation, so the model accuracy was not ideal. This explains that the fusion model generally had better prediction accuracy than the single monitoring technology.

### 3.2. Single monitoring technology to establish a prediction model

**Fig 11:**
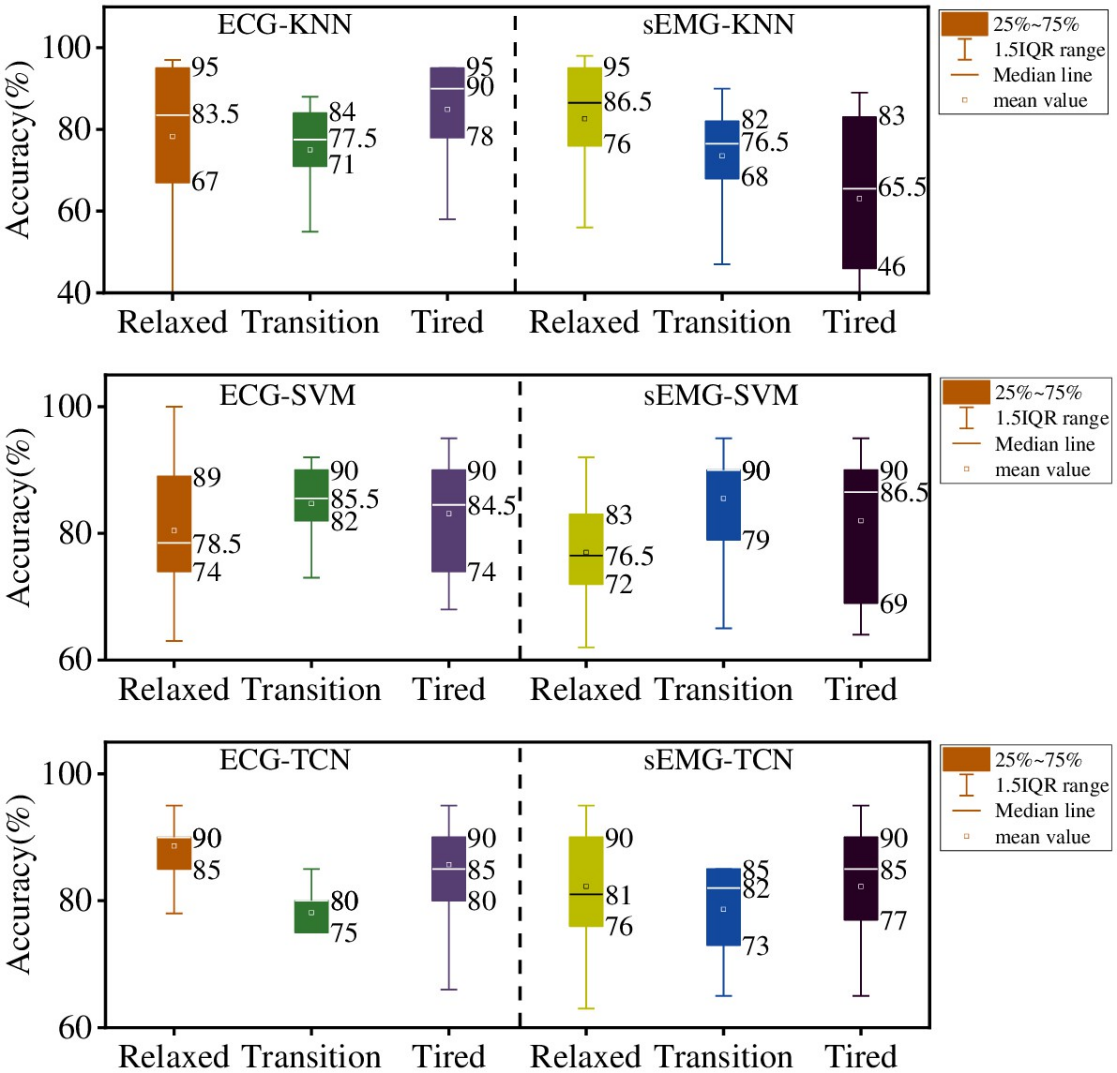
The accuracy statistics of each tester in the models.

In order to further analyze the causes of the results, the prediction models SEMG-TCN, ECG-TCN, SEMG-KNN and SEMG-SVM were established by sEMG and ECG single monitoring respectively. ECG—KNN, ECG—SVM. Because the prediction effect of the algorithm for each individual is not the same, the statistical accuracy result of each tester in the model is shown in Fig 11.

The difference between the six models was not huge. The accuracy of ECG-KNN was only 77.5% for transition, 83.5% for relaxed and 90% for fatigue. However, ECG-KNN was not good at resolving individual differences, with accuracy ranging from 95% to 67% for relaxed individuals and low outliers. This model is not suitable for any tester. The prediction accuracy of SEMG-KNN for the three states is 86.5%, 76.5% and 65.5% respectively. Compared with KNN algorithm, SVM budget model performance is more balanced, ECG-SVM prediction accuracy is 78.5%, 85.5%, 84.5%, the overall balance. However, the problem of outliers also exists. The lowest accuracy of ECG-SVM in predicting relaxed state is only 63%, while the lowest accuracy of SEMG-SVM in predicting three states is 62%, 65% and 64%, which is far lower than the highest accuracy (92%, 95% and 95%), indicating the poor stability of the model. The prediction accuracy of ECG-TCN and SEMG-TCN is average. The maximum accuracy of ECG-TCN was 93%, 85%, and 95% for the three states. The average accuracy was 86%, 78%, 85%, and the overall performance was good. But when it came to identifying fatigue, the lowest individual accuracy was only 70 percent. The maximum prediction accuracy of SEMG-TCN for the three states was 91%, 87% and 91%, and the average prediction accuracy was 81%, 82% and 85%. The results indicate that sEMG is more stable than ECG in fatigue prediction, and the prediction accuracy of three stages is more balanced. But the lowest individuals were only 68%, 67% and 74% accurate. In general, the six models have some predictive ability for fatigue state, and the sEMG-TCN and ECG-TCN models are better in terms of statistical average accuracy. However, all models are unstable, especially for the differences between individuals, so it is impossible to accurately predict each individual. Analysis showed that ECG was more sensitive in some subjects, while sEMG was more sensitive in others. Different stages vary greatly for different ECG or sEMG. Therefore, if you want to build a set of suitable fatigue detection system, you should start with a variety of monitoring methods. This was similar to the results obtained in literature [23].

### 3.3. Results of ECG and sEMG fusion prediction model

**Fig 12:**
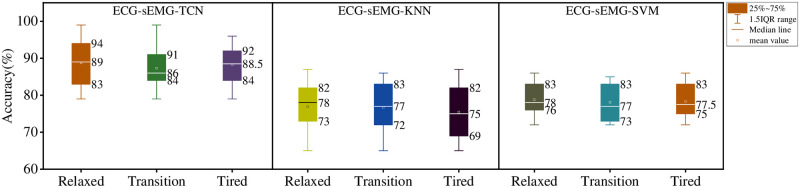
Prediction results of data fusion model.

The ECG-SEMG-TCN model was established by D-S evidence fusion method. ECG-sEMG-SVM and ECG-sEMG-KNN models were obtained by combining SVM and KNN with the same D-S evidence fusion method. The predicted results are shown in Fig 12.

Compared with the prediction results of ECG and sEMG fusion model and single monitoring model, the stability of the fusion model is better. Of the three models, the lowest predictive accuracy was found in the ECG-SEMG-KNN model (66%, 65%, 65%). There was no particularly low predictive accuracy. The maximum accuracy of ECG-sEMG-KNN is 87%, 86%, 87%. Note that this is considering the maximum and minimum values of individual differences, indicating that the fusion model is less affected by individual differences. The mean values of ECG-sEMG-KNN were 78%, 77%, 75%, and the overall model performance was poor among the three models. The ECG-sEMG-SVM model performed well with average accuracy of 78%, 77% and 77.5%, respectively. At the same time, the range of accuracy is further reduced, the maximum value is 85%~86%, the minimum value is 72%~74%, and the range of the three states is about 14.86% of the minimum value, which further improves the stability and robustness of the model. Of the three models, the ECG-sEMG-TCN model performed best. The average accuracy of prediction is 89%, 86% and 88.5% respectively, and the range of this model is also the smallest among the three models, which is only 11%, 7% and 8%. The model has strong generalization ability and stability.

In order to intuitively display the advantages and disadvantages of each model, precision, recall and F-Score are listed in Table 1. F-score is introduced to comprehensively evaluate accuracy and recall. The precision rate and recall rate are given the same weight in the calculation formula of F-score, so the performance of the model can only be compared with F-score index. According to the following results, ECG and sEMG fusion model had higher precision, recall rate and F-score than single technique for relaxed, transition and fatigue states. In the fusion model, TCN algorithm improved fatigue recognition performance, and its precision, recall rate and F-score were all higher than KNN and SVM models.

**Table 1:** The precision recall and F-score of three fatigue states.

model	Relaxed			Transition			Tired		
	Precision	Recall	F-score	Precision	Recall	F-score	Precision	Recall	F-score
ECG-KNN	0.8046	0.7580	0.8054	0.7780	0.7656	0.7915	0.8200	0.8492	0.8496
sEMG-KNN	0.8123	0.7384	0.7833	0.7682	0.7734	0.7634	0.8122	0.8352	0.8322
ECG-SVM	0.7623	0.7832	0.8140	0.7583	0.7820	0.7850	0.8231	0.7940	0.7931
sEMG-SVM	0.7380	0.8422	0.8163	0.7022	0.7219	0.7101	0.7108	0.7011	0.7599
ECG-TCN	0.8381	0.8204	0.8589	0.8254	0.8483	0.8669	0.8403	0.8970	0.9035
sEMG-TCN	0.8219	0.8122	0.8452	0.8123	0.8359	0.8511	0.8311	0.8540	0.8911
ECG-sEMG-TCN	0.9055	0.9494	0.9269	0.9303	0.9570	0.9435	0.8911	0.8764	0.8837
ECG-sEMG-KNN	0.9052	0.8988	0.9020	0.9300	0.9376	0.9338	0.8861	0.8721	0.8790
ECG-sEMG-SVM	0.8827	0.8721	0.8774	0.8976	0.8844	0.8910	0.8962	0.9003	0.8982

## 4. Conclusion

Current ECG and sEMG analysis and recognition often use time-domain and frequency-domain analysis methods to extract the local characteristics of signals, and then use classical machine learning algorithm to establish classification model, which can not fully reflect the time-domain morphology of muscles under the state of motion to a certain extent is not good for human physiological information mining. Therefore, a fatigue prediction method combining ECG and sEMG time series with TCN is proposed in this paper. By comparing the proposed method with the commonly used single monitoring technique based on time domain sequence, it is found that the fused prediction model and TCN has better performance, which has been mentioned in [24]. The application of fusion technology is indeed widely used in smart wearable devices, the difference lies in the established recognition algorithm.

TCN algorithm is widely used in gesture recognition, prosthesis control, rehabilitation and other fields, but the fusion of sEMG and ECG time series has not been reported. In this paper, several ECG and sEMG time series models are established, and several prediction algorithms including KNN, SVM and TCN, two common machine learning classifiers are compared. These machine learning classifiers all need to extract typical signal features, such as RMS, ZC, ARV, etc., and there are many related research reports. The results showed that ECG and sEMG fusion was improved in KNN, SVM and TCN classification, but individual differences caused by individual modeling methods still existed, and the model could not be guaranteed to be suitable for all participants. The results of ECG and sEMG model based on D-S evidence theory show that TCN has better classification performance than KNN and SVM, and the classification accuracy, recall rate and F-score are improved, and the lowest recognition accuracy of individuals is also higher than those of the two methods. The average precision of TCN (90.90%) and the average accuracy (88.0%) of TCN was also higher than that of previous studies [25] This provides a new idea for the integration of ECG and sEMG. Future studies will explore the effects of improved TCN or receptive field and model depth on recognition accuracy.
